# Editorial: Silicon-Based Nanomaterials: Synthesis, Optimization and Applications

**DOI:** 10.3389/fchem.2022.961641

**Published:** 2022-07-06

**Authors:** Lin Sun, Meipin Liu, Yuxiang Hu

**Affiliations:** ^1^ Key Laboratory for Advanced Technology in Environmental Protection of Jiangsu Province, School of Chemistry and Chemical Engineering, Yancheng Institute of Technology, Yancheng, China; ^2^ Jiangxi Key Laboratory of Function of Materials Chemistry, College of Chemistry and Chemical Engineering, Gannan Normal University, Ganzhou, China; ^3^ Key Laboratory of Advanced Functional Materials of Education Ministry of China, Faculty of Engineering and Manufacturing, Beijing University of Technology, Beijing, China

**Keywords:** silicon, preparation, energy storage, application, energy chemistry

Silicon (Si), the second most abundant element on earth crust, is rapidly gaining attention in life sciences (e.g., *in vivo* disease diagnosis and photothermal therapy), as well as the field of energy storage and conversion [such as lithium-ion batteries (LIBs) and solar cells] due to the biocompatibility, good luminescence, and the high energy density (Xu et al., 2018). As is well known, LIBs with Si anodes deliver a theoretically high specific capacity of ∼4,200 mAh g^−1^, which is significantly larger than that of commercial graphite anodes (372 mAh g^−1^). However, the large volume changes of Si during charge/discharge process and the complex preparing strategies severely hinder the practical applications (Sun et al., 2022).

**FIGURE 1:**
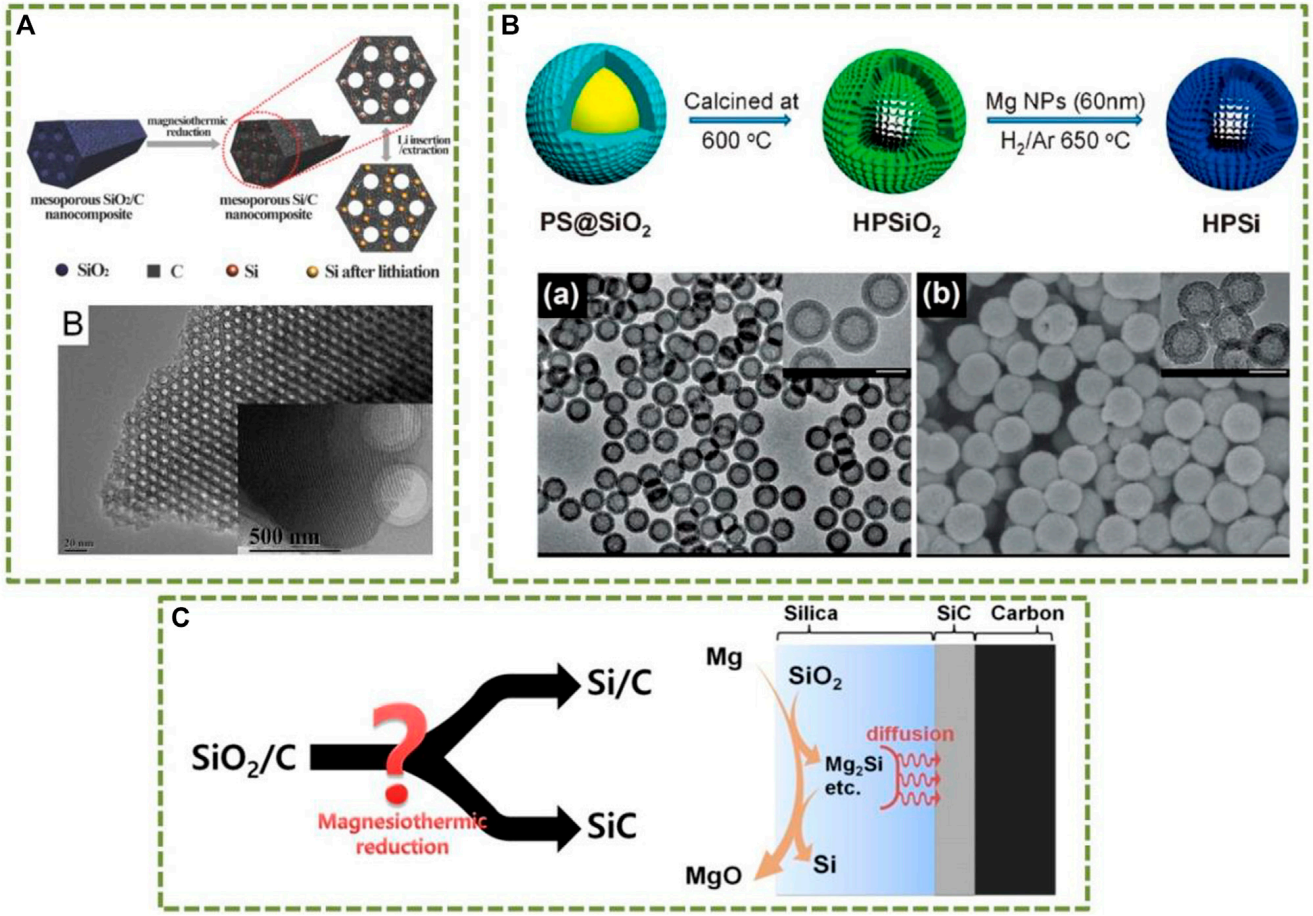
Representative works for the production of Si nanomaterials with magnesium thermal reduction **(A)** (Zhang et al., 2014); **(B)** (Chen et al., 2012) and **(C)** Ahn et al., 2016. Reprinted with permission. Copyright ^©^ 2014, 2012 WILEY-VCH Verlag GmbH and 2016 American Chemical Society.

The existing methods for synthesizing functional Si nanomaterials can usually be divided into two categories, that is “top-down” and “bottom-up” methods. The former strategy usually includes high temperature thermal reduction (e.g., carbon and magnesium thermal reduction), and electrochemical or chemical etching (Yuda et al., 2021). Magnesium thermal reduction is based on the interaction between the magnesium vapor and the SiO_2_ precursor to afford Si through gas-solid reaction. In general, the replica of Si with the same morphology as SiO_2_ precursors can be obtained by controlling the reaction temperature, flowing gas rate and some other reaction parameters (Sun et al., 2017). As illustrated in Figure 1, some representative works related to the magnesium thermal reduction method are presented. Figures 1A,B show the conventional magnesium thermal reduction method to afford Si replicas from SiO_2_ precursors (Chen et al., 2012; Zhang et al., 2014). However, the direct magnesium thermal reduction of SiO_2_/C nanocomposite is extremely easy to form byproducts, such as Mg_2_Si and SiC. Ahn et al. proposed a formation mechanism of Si and SiC by magnesiothermic reduction of SiO_2_/C, as shown in Figure 1C. SiC is formed at the interface between SiO_2_ and carbon when silicon intermediates, mainly in situ-formed Mg_2_Si, encounter carbon through diffusion. Otherwise, Si is formed, which is supported by an *ex-situ* reaction between Mg_2_Si and carbon nanosphere that results in SiC (Ahn et al., 2016).

Electrochemical and chemical etching (HF/H_2_O_2_ or HF/metal-assisted system) generally start from bulk Si to realize the morphology controllable of Si *via* the regulation of reaction parameters, such as the applied current density, the HF concentration, and the reaction time (Huo et al., 2020). In general, these methods have been widely used in photovoltaic industry, however, the environmental issue of strong acid and base system should be taken into account. On the other hand. the “bottom-up” methods generally include chemical vapor deposition (CVD), the classical vapor-liquid-solid (VLS) growth, the reduction of high valent Si (Sun et al., 2019). The preparation of Si by CVD methods generally uses volatile silicon sources such as SiH_4_ and SiCl_4_ as the feed stock and the targeted Si is produced by the decomposition of Si precursors under high temperature conditions. Concurrently, Si nanomaterials with various sizes can be obtained by adjusting the types of precursors, the reaction temperature, and the flowing carrier gas rate. Additionally, one-dimensional (1D) Si nanowires can be obtained by vapor-liquid-solid (VLS) growth, that is, the solid solution derived from Si precursors are formed on the surface of metal catalysts. When Si is saturated in the solid solution, 1D Si nanowires with specific shapes are produced in a particular direction (Puglisi et al., 2019). Moreover, zero-dimensional (0D) Si quantum dots can generally be reduced from high valent Si compounds, and the reducing agents can be metallic Na, K or sodium naphthalene solution, LiAlH_4_ (Na et al., 2019).

It is worth considering that the current existing synthetic methods of Si nanomaterials have considerable disadvantages of high energy consumption, low yield, harsh reaction conditions and difficult to scale production. As is known to all, the “bottom-up” wet chemical synthesis of nanomaterials has the merits of simple operation, easy amplification and the controllable morphology. However, different from the preparation of metals or metal oxides, Si precursors that can ionize in solvents are very scarce. Although the Zintl phase compounds of Si, such as Na_4_Si_4_ and K_4_Si_4_, can dissociate from Si_4_^4-^ ion clusters in liquid ammonia at −70°C, such harsh conditions are restrictive to realize the scaled-up applications (Schiegerl et al., 2018). Therefore, it is one of the most important directions to explore new Si precursors that are suitable for wet chemistry under mild conditions. In this topic collection, advances of synthesis methods for porous Si and Si nanocrystals are summarized, meanwhile, some biomass derived Si nanomaterials are reported. In addition, the various applications of functional Si-based nanomaterials, such as energy storage, photoluminescent, catalysis, are also included.

We hope it will be helpful for readers to further understand the preparation and application of advanced silicon nanomaterials.
